# TIPARP is involved in the regulation of intraocular pressure

**DOI:** 10.1038/s42003-022-04346-0

**Published:** 2022-12-19

**Authors:** Youjia Zhang, Maomao Song, Yingwen Bi, Yuan Lei, Xinghuai Sun, Yuhong Chen

**Affiliations:** 1 Department of Ophthalmology & Visual Science, Eye & ENT Hospital, Shanghai Medical College, Fudan University, Shanghai, 200031 China; 2 NHC Key Laboratory of Myopia, Chinese Academy of Medical Sciences, and Shanghai Key Laboratory of Visual Impairment and Restoration, Fudan University, Shanghai, 200031 China; 3 Department of Pathology, Eye & ENT Hospital, Shanghai Medical College, Fudan University, Shanghai, 200031 China; 4 State Key Laboratory of Medical Neurobiology and MOE Frontiers Center for Brain Science, Institutes of Brain Science, Fudan University, Shanghai, 200032 China

**Keywords:** Glaucoma, Pathogenesis, Molecular biology

## Abstract

Elevated intraocular pressure (IOP) is the major risk factor for glaucoma. The molecular mechanism of elevated IOP is unclear, which impedes glaucoma therapy. 2,3,7,8-tetrachlorodibenzo-p-dioxin (TCDD)-inducible Poly-ADP-ribose Polymerase (TIPARP), a member of the PARP family, catalyses mono-ADP-ribosylation. Here we showed that TIPARP was widely expressed in the cornea, trabecular meshwork, iris, retina, optic nerve, sclera, and choroid of human eyes. The expression of TIPARP was significantly upregulated in the blood and trabecular meshwork of patients with primary open angle glaucoma compared with that of healthy controls. Transcriptome analysis revealed that the expression of genes related to extracellular matrix deposition and cell adhesion was decreased in *TIPARP*-upregulated human trabecular meshwork (HTM) cells. Moreover, western blot analysis showed that collagen types I and IV, fibronectin, and α-SMA were increased in *TIPARP*-downregulated or TIPARP-inhibited HTM cells. In addition, cross-linked actin networks were produced, and vinculin was upregulated in these cells. Subconjunctival injection of the TIPARP inhibitor RBN-2397 increased the IOP in *Sprague–Dawley* rats. Therefore, we identified TIPARP as a regulator of IOP through modulation of extracellular matrix and cell cytoskeleton proteins in HTM cells. These results indicate that TIPARP is a potential therapeutic target for ocular hypertension and glaucoma.

## Abstract (web summary)

TIPARP, an inducible poly-ADP-ribose polymerase, is identified as a novel regulator of intraocular pressure through modulation of extracellular matrix and cell cytoskeleton proteins in trabecular meshwork cells.

## Introduction

Glaucoma is a group of diseases characterized by progressive degeneration of the optic nerve and is the top reason for irreversible blindness worldwide^1^. Primary open angle glaucoma (POAG) is the most common type of glaucoma and is a complex disease with genetic heterogeneity^1^. Elevated intraocular pressure (IOP) is the major and only modifiable risk factor for POAG^1,2^. IOP is determined by the balance between aqueous humor secretion and drainage mainly through the conventional outflow pathway consisting of the trabecular meshwork, juxtacanalicular tissue and Schlemm’s canal^2,3^. Abnormally high resistance to aqueous humor outflow induced by trabecular meshwork malfunction is an important aetiology of POAG^4–6^. However, the underlying molecular and cellular mechanisms of increased resistance to aqueous humor outflow still need to be explored.

TIPARP (TCDD-inducible poly-ADP-ribose polymerase), also known as PARP7 or ARTD14, is a TCDD (2,3,7,8-tetrachlorodibenzo-p-dioxin)-induced poly-ADP-ribose polymerase (PARP)^7^. As a member of the PARP family, TIPARP transfers mono-ADP-ribose from nicotinamide adenine dinucleotide (NAD^+^) to substrates and catalyses mono-ADP-ribosylation, which is an important and ubiquitous post-translational protein modification^8^. TIPARP has important functions in many physiological processes, including gene regulation, viral response, and cytoskeleton regulation^9^. The dysregulation of TIPARP may induce neuronal developmental disorders, abnormal antiviral responses, some types of cancer, and dioxin-induced steatohepatitis^10–16^. Evidence of the association between TIPARP and POAG has been reported in a few studies. A meta-analysis of more than 13,000 Europeans revealed that the SNP rs9822953 located in *TIPARP* is significantly associated with central corneal thickness, which is a risk factor for POAG^17^. In a glaucomatous lamina cribrosa cell model induced by hypoxia stress, the expression of *TIPARP* was significantly increased^18^. However, the role and regulation of TIPARP in trabecular meshwork cells and aqueous humor outflow are unknown.

To obtain greater insights into the relationship between TIPARP and IOP regulation, we examined TIPARP expression in patients with POAG and explored the function and molecular mechanism of TIPARP in aqueous humor outflow regulation. This study suggested that TIPARP participated in IOP regulation by modulating extracellular matrix (ECM) deposition, cell adhesion and cross-linked actin network (CLAN) formation.

## Results

### Expression and distribution of TIPARP in human eyes

**Fig. 1:**
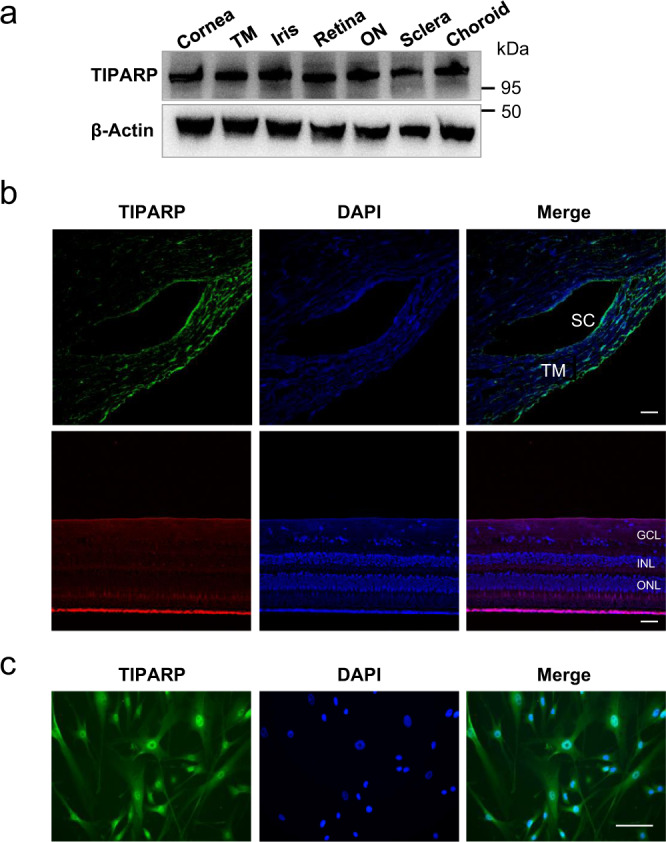
Expression and distribution of TIPARP in human trabecular meshwork tissues and cells. **a** TIPARP expression in the human cornea, trabecular meshwork, iris, retina, optic nerve, sclera, and choroid by Western blot. **b** Immunofluorescence of TIPARP showed positive staining in the human trabecular meshwork, Schlemm’s canal and retina. Scale bar = 25 μm. **c** In human trabecular meshwork cells, immunofluorescence staining of TIPARP indicated nuclear and cytoplasmic localization. Scale bar = 100 μm. TM trabecular meshwork, ON optic nerve, SC Schlemm’s canal, GCL ganglion cell layer, INL inner nuclear layer, ONL outer nuclear layer.

Western blot analysis showed that TIPARP was expressed in the human cornea, trabecular meshwork, iris, retina, optic nerve, sclera, and choroid (Fig. 1a). Further immunofluorescence staining confirmed that TIPARP was expressed in human aqueous humor outflow tissues and retina (Fig. 1b). Human trabecular meshwork (HTM) cell was confirmed and characterized by a classical method^19^. After 5 days of treatment with dexamethasone, the myocilin expression of HTM cells was increased according to immunofluorescence (Supplementary Fig. 1a) and Western blot analyses (Supplementary Fig. 1b). In HTM cells, TIPARP was expressed in both the nuclei and cytoplasm of the cells (Fig. 1c).

### TIPARP was upregulated in patients with POAG and mouse eyes after perfusion

**Fig. 2:**
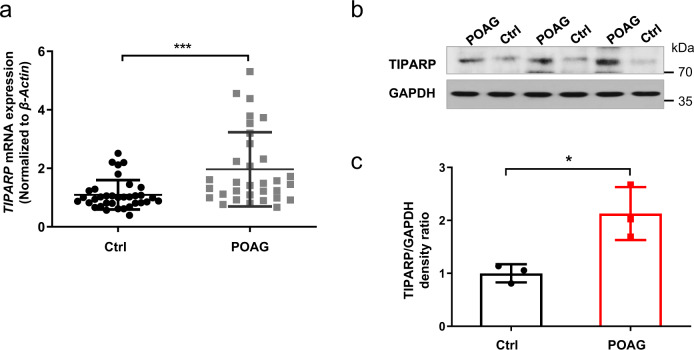
TIPARP expression in patients with POAG. **a** The expression of *TIPARP* was significantly higher in the blood samples of the patients with POAG (*n* = 32) than in the controls (*n* = 35) (1.80-fold, *P* < 0.001, Mann‒Whitney U test). **b**, **c** Western blot analysis showed that the TIPARP protein expression was significantly higher in the surgical trabecular meshwork from the patients with POAG than in the human donor eyes (2.13-fold, *n* = 3, *P* = 0.02, *t*-test). Data are presented as the means ± standard deviations. *P < 0.05, ***P < 0.001.

In blood samples, the *TIPARP* level in the patients with POAG (*n* = 32) was significantly higher than that in the controls (*n* = 35) (1.80-fold, *P* < 0.001, Fig. 2a). In the trabecular meshwork tissues, Western blot analysis showed that TIPARP was upregulated by 2.13-fold in the patients with POAG (*n* = 3) compared with that in the control human donor eyes (*n* = 3, *P* = 0.02, Fig. 2b, c).

To further investigate TIPARP expression changes under elevated IOP, we perfused mouse eyes ex vivo at constant pressure for 4 h. Western blot analysis showed that the expression of TIPARP in outflow tissues was upregulated in 18 mmHg perfusion compared with that in 6 mmHg perfusion (*n* = 3 in the 6 mmHg group and *n* = 4 in the 18 mmHg group, *P* = 0.018, Supplementary Fig. 2a and 2b).

### RNA-seq analysis of *TIPARP*-overexpressing HTM cells

**Fig. 3:**
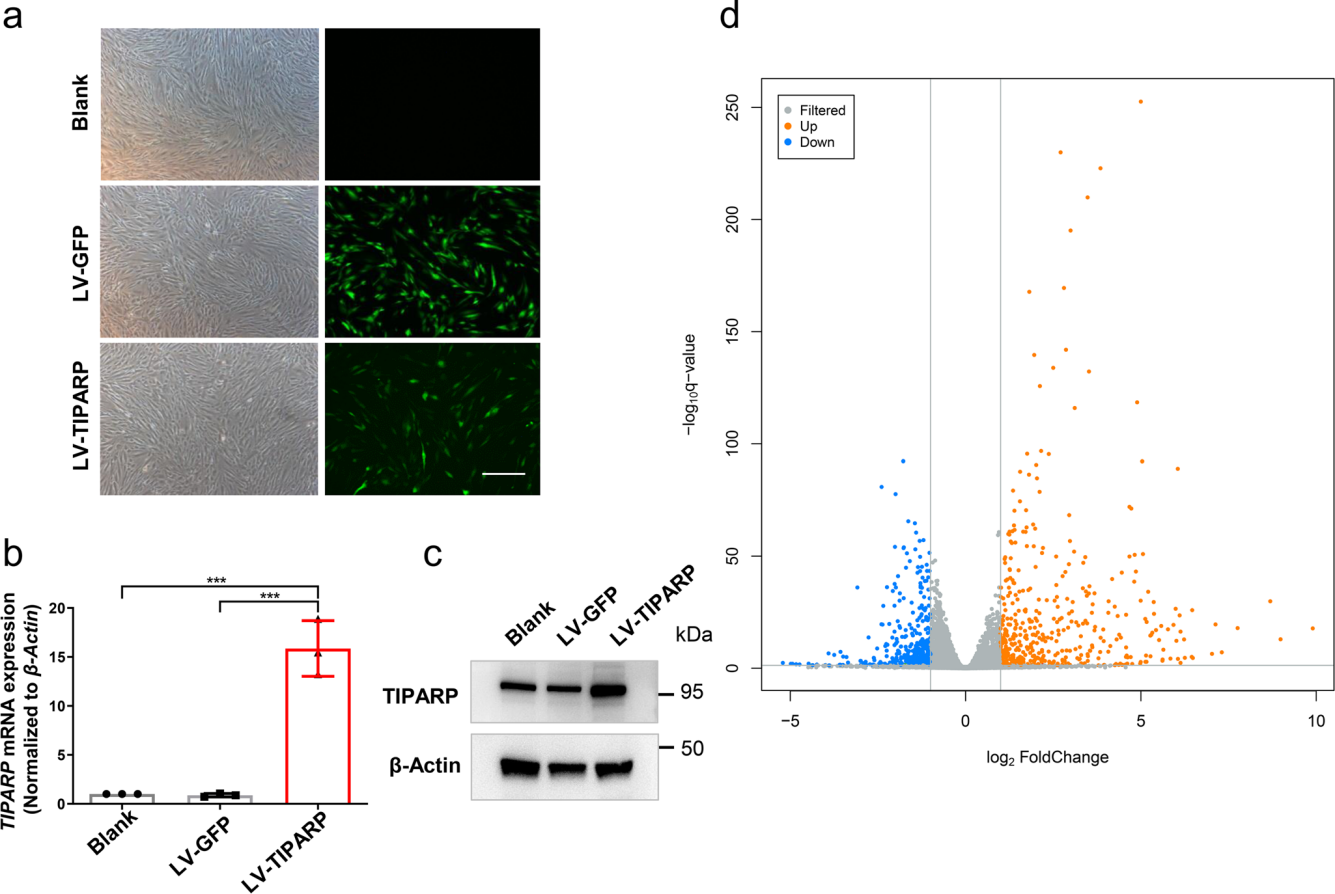
Transcriptome analysis of *TIPARP* overexpressing HTM cells. **a** Bright-field and fluorescence micrographs of HTM cells transduced with TIPARP encoding lentivirus (LV-TIPARP) and scrambled lentivirus (LV-GFP). Green fluorescence indicated successful transduction in the cell. The transduction efficient was 57.50% ± 1.94% in LV-GFP group and 50.21% ± 1.50% in LV-TIPARP group. **b** qRT‒PCR showed that LV-TIPARP transduction significantly increased *TIPARP* expression compared with that of blank cells and LV-GFP cells (*n* = 3, *P* < 0.001). **c** Western blot confirmed that LV-TIPARP transduction increased TIPARP protein expression compared with that in the LV-GFP cells. **d** Volcano plot showing 499 upregulated and 390 downregulated genes in the LV-TIPARP cells compared with the LV-GFP cells in total (fold change >1.5 or <0.6, *q* value < 0.05, *n* = 3 independent replicates for each group). Data are presented as the means ± standard deviations. ***P < 0.001. Scale bar = 50 μm.

**Fig. 4:**
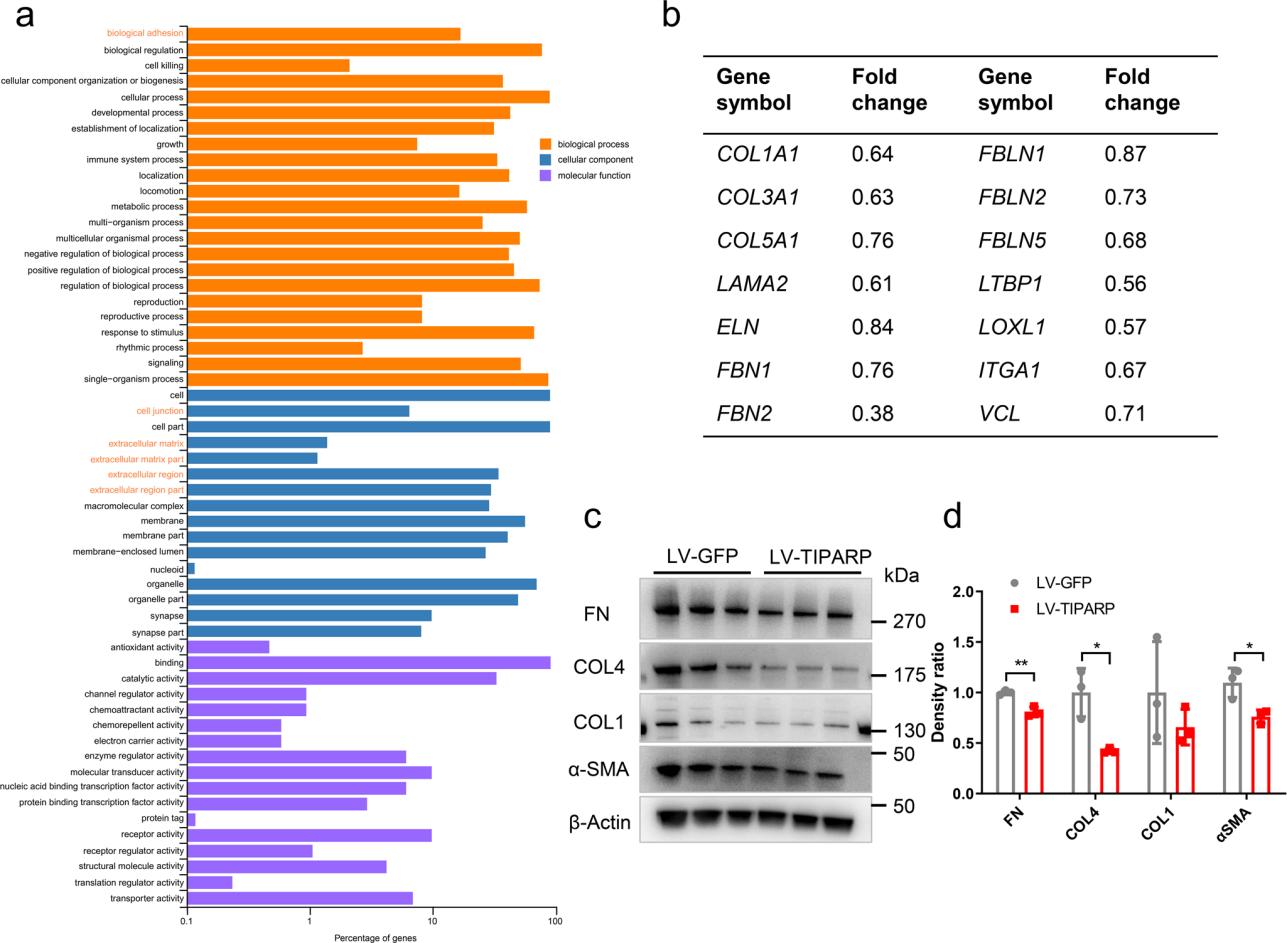
Identification and verification of the expression of genes related to the conventional outflow function identified by the transcriptome data. **a** The differentially expressed mRNAs (*q* value <0.05, fold changes >1.5 or <0.6) were classified by Gene Ontology terms. **b** Genes related to the conventional outflow function and its expression in transcriptome data. **c**, **d** Western blot and quantitative analyses showed fibronectin, collagen type IV, and α-SMA expression decreased in the LV-TIPARP-treated HTM cells compared with the LV-GFP control cells (*n* = 3, *t* test). Data are presented as the means ± standard deviations. *P < 0.05, **P < 0.01. COL4 collagen type IV, COL1 collagen type I, FN fibronectin.

RNA-seq was performed in *TIPARP*-overexpressing HTM cells to investigate the possible downstream transcriptome changes upon *TIPARP* upregulation. Green fluorescence indicated the transduction efficient was 57.50% ± 1.94% in HTM cells transduced with scrambled lentivirus (LV-GFP HTM cells) and 50.21% ± 1.50% in HTM cells transduced with *TIPARP*-encoding lentivirus (LV-TIPARP HTM cells) (Fig. 3a). The *TIPARP* mRNA and protein expression levels were significantly increased in the LV-TIPARP HTM cells compared with that in the HTM cells treated without lentivirus (blank cells) and LV-GFP HTM cells (Fig. 3b, *n* = 3 lines, *P* < 0.001, and Fig. 3c). Transcriptome analysis identified 499 upregulated and 390 downregulated genes in the LV-TIPARP HTM cells compared with the LV-GFP HTM cells (Fig. 3d, *n* = 3 lines). The Gene Ontology biological process analysis of the differentially expressed genes revealed that biological adhesion, cell junction, ECM, and extracellular region were associated with *TIPARP* upregulation (Fig. 4a). Specifically, as a result of upregulation of *TIPARP* mRNA, the expression of a series of genes encoding ECM components was reduced in the LV-TIPARP HTM cells, including collagen genes (e.g., *COL1A1*, *COL3A1*, and *COL5A1*), laminins (e.g., *LAMA2*), elastin and fibrillin (e.g., *ELN* and *FBN1-2*), fibulins (e.g., *FBLN1*, *FBLN2*, and *FBLN5*), and microfibril-related genes (e.g., *LOXL1-3* and *LTBP1-4*). Genes participating in cell-cell and cell-ECM adhesion (e.g., *VCL* and *ITGA1*) were reduced in the LV-TIPARP HTM cells (Fig. 4b). In addition, the expression of most tissue inhibitor of metalloprotease family members was downregulated (*TIMP1*, *TIMP3*, and *TIMP4*). However, the expression of different members of the matrix metalloproteinase family did not show the same trend. Western blot analysis confirmed the downregulation of collagen type IV, fibronectin and α-SMA in the LV-TIPARP HTM cells (Fig. 4c, d).

### Inhibition of TIPARP enhanced ECM deposition and HTM cell contraction

**Fig. 5:**
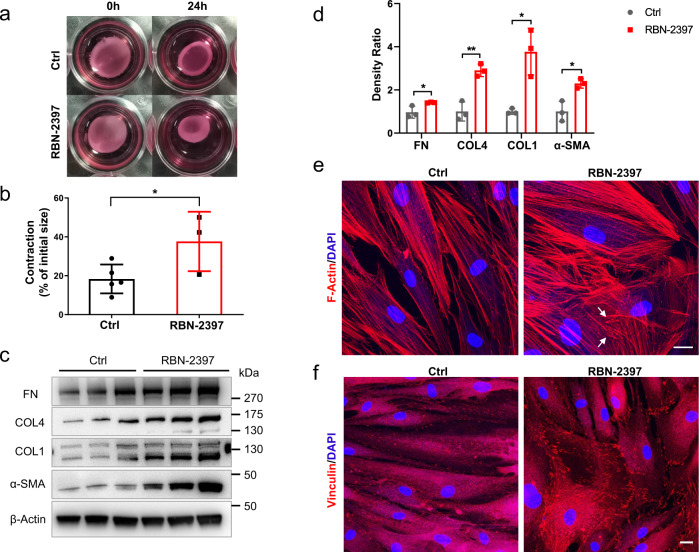
The TIPARP inhibitor RBN-2397 enhanced HTM cell contraction, ECM deposition, CLAN formation and focal adhesion. **a**, **b** HTM cell contractility was measured after 24 h with or without RBN-2397 (1 μM) exposure. Contraction data are presented as the percent of the initial size (means ± standard deviations). RBN-2397 exposure enhanced HTM cell contraction compared to that of the control cells. (*n* = 3 in the RBN-2397 group and *n* = 5 in the control group, *t* test). **c**, **d** Western blot and quantitative analyses showed that the expression of fibronectin, collagen type IV, collagen type I and α-SMA increased in RBN-2397-treated HTM cells compared with the control cells (*n* = 3, *t* test). **e** After 48 h of RBN-2397 treatment, HTM cells increased the formation of CLAN (white arrows), as shown by phalloidin staining, compared to that of the control cells. **f** The RBN-2397-treated HTM cells exhibited an increase in the focal adhesion marker vinculin by immunofluorescence compared to the control cells. Data are presented as the means ± standard deviations. *P < 0.05, **P < 0.01. Scale bar = 20 μm. CLAN cross-linked actin network, COL1 collagen type I, COL4 collagen type IV, FN fibronectin.

Following the TIPARP overexpression experiments, the molecular consequence of TIPARP inhibition was studied. RBN-2397 is a potent and selective small-molecule inhibitor of TIPARP catalytic function^15^. The cocrystal structure of RBN-2397 showed that it can bind to the NAD^+^-binding pocket^15^. RBN-2397 treatment did not alter the expression level of TIPARP in HTM cells (Supplementary Fig. 3). The 1 μM RBN-2397-treated HTM cells contracted 37.66 ± 8.81 percent of their initial size (*n* = 3), while the control cells contracted 18.33 ± 3.32 percent of their initial size after 24 h of treatment (*n* = 5) (*P* = 0.048, Fig. 5a, b). Western blot analysis revealed that the expression of collagen type I, collagen type IV, fibronectin, and α-SMA in HTM cells was markedly increased after 1 μM RBN-2397 treatment compared to the controls (*n* = 3, all *P* < 0.05, Fig. 5c, d). Moreover, phalloidin labeling of F-actin showed that 1 μM RBN-2397 significantly induced the formation of CLAN structures after 48 h of treatment (25.1% ± 1.6% in the RBN-2397 group vs. 11.0% ± 1.1% in the control group, *n* = 3, *P* = 0.002, Fig. 5e). Immunofluorescence staining of vinculin showed an increase in focal adhesion after RBN-2397 treatment (Fig. 5f). These results suggested that TIPARP participates in HTM contraction and ECM deposition by regulating mono-ADP-ribosylation.

### TIPARP knockdown increased ECM protein expression

**Fig. 6:**
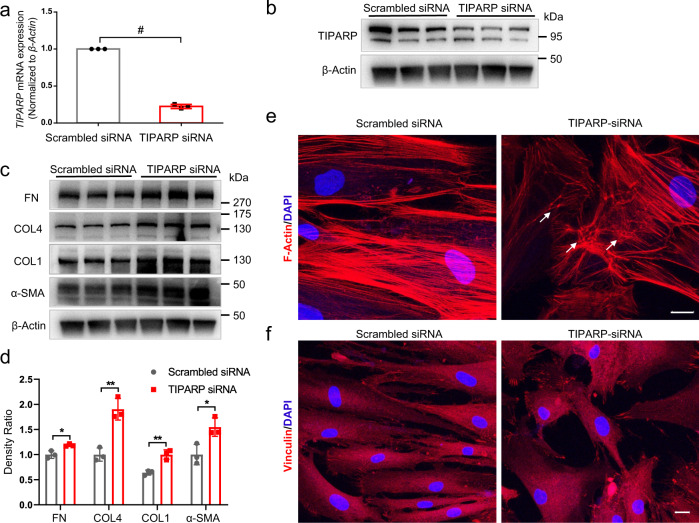
Suppression of *TIPARP* expression by siRNA transfection activated the formation of CLAN, focal adhesion, and ECM deposition in HTM cells. HTM cells were transfected with *TIPARP* siRNA or scramble siRNA. **a** qRT‒PCR showed that *TIPARP* siRNA transfection decreased *TIPARP* expression by 0.22-fold compared with scrambled siRNA transfected controls (*n* = 3, *t* test). **b** Western blot confirmed that the expression of TIPARP protein was downregulated in the *TIPARP* siRNA-treated HTM cells compared with the scramble siRNA-treated HTM cells. **c**, **d** Western blot and quantitative analyses showed that the expression of fibronectin, collagen type IV, collagen type I and α-SMA increased in the *TIPARP* siRNA-treated HTM cells compared with the scrambled control cells (*n* = 3, *t* test). **e** The HTM cells treated with *TIPARP* siRNA showed increased formation of CLAN (white arrows) by phalloidin staining compared to the scrambled siRNA-treated control cells. **f** The HTM cells treated with *TIPARP* siRNA exhibited an increase in the focal adhesion marker vinculin by immunofluorescence compared to the control cells. Data are presented as the means ± standard deviations. *P < 0.05, **P < 0.01, ^#^*P* < 0.0001. Scale bar = 20 μm. CLAN cross-linked actin network, COL1 collagen type I, COL4 collagen type IV, FN fibronectin.

To further demonstrate the pathological effect of TIPARP inhibition, we knocked down *TIPARP* expression using *TIPARP* small interfering RNA (siRNA) in HTM cells. The mRNA and protein expression levels were decreased by treatment with *TIPARP* siRNA compared with the scrambled siRNA control (Fig. 6a, b). The HTM cells with *TIPARP* knockdown exhibited a marked increase in the expression of collagen type I, collagen type IV, fibronectin, and α-SMA compared to the control cells (Fig. 6c, d, all *P* < 0.05), which indicated excessive ECM deposition and pathologic profibrotic responses under TIPARP deficiency. Moreover, following downregulation of *TIPARP* expression, the formation of CLAN structures increased, which could increase the stiffness of HTM cells (39.3% ± 4.0% in the *TIPARP* siRNA group vs. 18.6% ± 1.3% in the scrambled siRNA group, *n* = 3, *P* = 0.008, Fig. 6e). In addition, the *TIPARP*-deficient HTM cells showed an increase in focal adhesions based on vinculin immunofluorescence staining (Fig. 6f). Taken together, these observations revealed that TIPARP regulates ECM production and cell adhesive properties in HTM cells.

### A TIPARP inhibitor increased IOP in rats

**Fig. 7:**
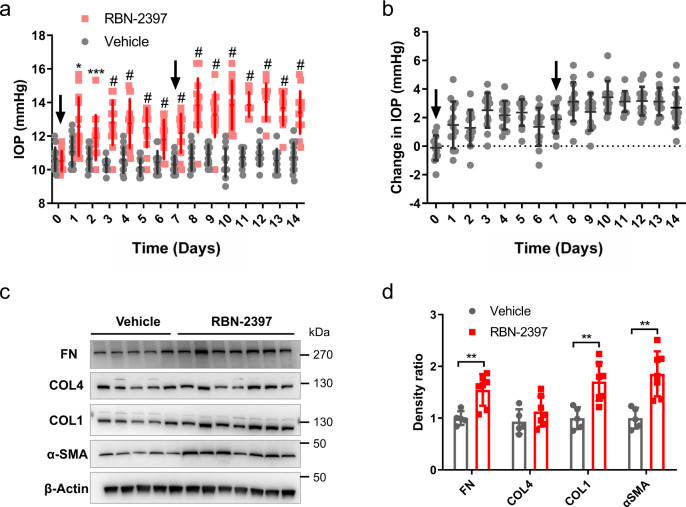
Effects of the TIPARP inhibitor RBN-2397 on IOP in vivo. **a** A total 50 μL of 10 μM RBN-2397 was injected into the upper and lower subconjunctival space of *Sprague–Dawley* rat eyes on Day 0 and Day 7 (black arrows). IOPs were increased after RBN-2397 subconjunctival injection compared with that of the vehicle control group (*n* = 13 eyes in each group). **b** Changes in IOPs due to RBN-2397 injection over time of the experiment compared to those of the vehicle control group are shown. **c**, **d** Western blot and quantitative analyses showed that the expression of fibronectin, collagen type I and α-SMA increased in outflow tissue after RBN-2397 injection (*n* = 7 in the RBN-2397 group and *n* = 5 in the vehicle control group). Data are presented as the means ± standard deviations. *P < 0.05, **P < 0.01, ***P < 0.001, ^#^*P* < 0.0001. COL1 collagen type I, COL4 collagen type IV, FN fibronectin.

To further assess the effect of TIPARP inhibition in vivo, we measured IOPs in rat eyes after RBN-2397 injection. A total 50 μL of 10 μM RBN-2397 or vehicle (0.1% dimethylsulfoxide) was injected into the subconjunctival space of *Sprague–Dawley* rat eyes on Day 0 and Day 7. From Day 1 to Day 7, the IOPs of the RBN-2397-treated eyes significantly increased compared to those of the vehicle-treated eyes (*n* = 13, all *P* <  0.05). After the second injection on Day 7, the IOPs of the RBN-2397-treated group increased steadily higher than those of the vehicle control group from Day 8 to Day 14 (*n* = 13, all *P* < 0.0001) (Fig. 7a, b). On Day 14, outflow tissue was collected for Western blot analysis. RBN-2397 subconjunctival injection caused ECM deposition verified by the upregulation of collagen type I, fibronectin, and α-SMA (Fig. 7c, d).

## Discussion

In the present study, we identified a key role for TIPARP in the regulation of trabecular meshwork function and IOP. We investigated the expression of TIPARP in the eye, and found that the TIPARP expression levels were increased in the trabecular meshwork and blood in the patients with POAG compared with the controls. These findings indicate the critical role of TIPARP in IOP regulation. Then, we showed that TIPARP participated in the regulation of ECM components and cell-ECM adhesion by utilizing *TIPARP*-upregulated HTM cells. We demonstrated that loss of TIPARP function caused ECM deposition, CLAN formation and focal adhesion. In addition, we found that the inhibition of TIPARP resulted in elevated IOP in rats. In conclusion, our results show that TIPARP participates in the pathogenesis of POAG.

The PARP family is a group of enzymes catalysing cellular ADP-ribosylation, which is an important post-translational protein modification. ADP-ribosylation is involved in many biological processes, such as oxidative stress, DNA repair, cytoskeletal regulation, transcriptional regulation, and immune cell function^13,20^. Some PARPs, including PARP1, catalyse poly ADP-ribosylation and have been well studied^9^. Other PARPs, including TIPARP, also known as mono(ADP-ribosyl) transferase (MART) enzymes, catalyse mono-ADP-ribosylation (MARylation), which is not well understood^9^. As a MART enzyme, TIPARP catalyses the mono-ADP-ribosylation of target proteins and exerts effects on the viral response, transcriptional regulation, stem cell pluripotency, neuronal function and cytoskeleton regulation^9–14^. In addition, TIPARP has been identified as a potential target of cancer therapy^12,15,16^. Although the importance of TIPARP has been gradually discovered in recent years, research on its function remains very limited.

Our study focused on the role of TIPARP in IOP regulation. TIPARP is expressed in many mouse tissues, including the heart, brain, lung, liver, spleen, and reproductive organs^7^, but the distribution of TIPARP in the eye has yet to be described. We showed that TIPARP was expressed widely in eye tissues, including the cornea, trabecular meshwork, iris, sclera, choroid, retina, and optic nerve. In addition, we demonstrated that TIPARP expression was upregulated in patients with POAG and in eyes perfused ex vivo. The distribution of TIPARP in the ocular drainage structures and the abnormal expression of TIPARP in the patients with POAG suggested that TIPARP might play a regulatory role in maintaining trabecular meshwork function and normal IOP.

We further clarified the role and mechanism of TIPARP in IOP regulation through in vitro and in vivo experiments. The main glaucoma-related events occurring in trabecular meshwork include oxidative stress, mitochondrial impairment, endothelial dysfunction, and proteome changes in the aqueous humor, which includes mitochondrial proteins, cell adhesion proteins, protein kinases, and neuronal proteins^21^. Together, these proteins are involved in IOP regulation. The function of PARPs and the role of ADP-ribosylation in aqueous humor outflow are still unclear. We found that a variety of genes associated with ECM and focal adhesion were downregulated in the *TIPARP*-overexpressing HTM cells by RNA-seq. Previous studies have demonstrated that the trabecular meshwork regulates aqueous humor outflow resistance by cells and their ECM interplay and ECM protein interactions^5,6^. Abnormal changes in the ECM have dramatic effects on trabecular meshwork function^22,23^. The Gene Ontology analysis of RNA-seq identified ECM, ECM-related genes, biological adhesion, and cell junction as key pathways impacted by *TIPARP* upregulation. The expression of many ECM component genes, including collagen (*COL1A1*, *COL3A1*, and *COL5A1*), elastin and fibrillin (*ELN* and *FBN1-2*), laminins (*LAMA2*), and fibulins (*FBLN1-2* and *5*), was decreased. The ECM deposition regulatory genes, including *LTBP1* and *LOXL1*, and the cell adhesion genes *ITGA1* and *VCL* also showed decreased. In addition, the expression of most tissue inhibitor of metalloprotease family members was downregulated (*TIMP1*, *TIMP3*, and *TIMP4*), which was consistent with the expression changes in ECM gene expression. Therefore, TIPARP might be involved in the maintenance of IOP by regulating ECM deposition and cell adhesion. To further confirm the function of TIPARP, we inhibited and downregulated TIPARP in HTM cells. Consistent with the RNA-seq results, increased ECM deposition, cell adhesion, and CLAN formation were observed in the TIPARP-inhibited or downregulated HTM cells. Excessive ECM deposition has been observed in the POAG outflow pathway, which results in tissue stiffness and increases the resistance to aqueous humor outflow^5,21,24^. The actin cytoskeleton is another key factor in the regulation of outflow resistance. The abnormal arrangement of the cytoskeleton and formation of CLAN affect trabecular meshwork contraction and then influence outflow resistance^25^. In addition, an abnormal increase in α-SMA indicates pathologic fibrotic responses that produce myofibroblast-like contractile cells and increase tissue stiffness^26–28^. Then, the in vivo experiment provided further validation of TIPARP regulating IOP. After subconjunctival injection of the TIPARP inhibitor RBN-2397 in rats, the IOP of the rats was elevated. Taken together, these in vitro and in vivo experiments confirmed that TIPARP was involved in IOP regulation by regulating the ECM, actin cytoskeleton and cell adhesion.

Previous studies have also demonstrated that TIPARP and its mono-ADP-ribosylation are important targets for regulating the cell cytoskeleton and cell adhesion. In a study on ovarian cancer, knockdown of *TIPARP* was reported to reduce cell growth, migration, and invasion in OVCAR4 cells^12^. The functions of TIPARP target proteins identified by mass spectrometry were enriched in cell-ECM adhesion and cytoskeleton organization^12^. Similarly, *Tiparp*^–/–^ mice presented a decrease in neural progenitor cell proliferation and a reduction in neural stem cell migration because of the abnormal mono-ADP-ribosylation levels of cytoskeletal proteins^13^. Our results supported these studies about the roles of TIPARP in cell-ECM adhesion and the cell cytoskeleton and added additional roles of TIPARP in ECM deposition.

Interestingly, TIPARP expression was upregulated in blood samples and the trabecular meshwork of our patients with POAG. Furthermore, an ex vivo mouse eye perfusion model showed that elevated intraocular pressure could lead to the upregulation of TIPARP expression in outflow tissues of mouse eyes, which was consistent with the upregulation of TIPARP in patients with POAG. Combined with the results of a series of in vitro and in vivo experiments on TIPARP functions, we conclude that the increased expression level of *TIPARP* might play a positive role in IOP regulation in POAG. Previous studies have shown that a series of mechanisms exist to maintain IOP homeostasis^29^. For example, the levels of some matrix metalloproteinases, important ECM remodeling regulatory factors, are increased in the aqueous humor of patients with POAG^29–31^. Similarly, the upregulation of TIPARP could partially reduce the aqueous humor outflow resistance and help to re-establish the IOP balance. In the future, the development of agonists or analogs of TIPARP may be a new method to reduce IOP.

One of the deficiencies of our study is the comparability of trabecular meshwork tissues between patients with POAG and controls. Trabeculectomy for the patients with POAG was performed by an experienced ophthalmologist to ensure that trabecular meshwork tissue was included. The trabecular meshwork tissues from the control donor eyes were isolated using a similar method to ensure that the tissues of the two groups had similar compositions. The cadaveric trabecular meshwork tissues were dissected within 12 h after death from our eye bank. Prolonged time could affect the freshness of cadaveric trabecular meshwork tissues, which might affect the expression of TIPARP. Therefore, TIPARP expression in the blood of the patients with POAG and the controls was tested to provide additional evidence.

In conclusion, our study illustrated the key role of TIPARP in the regulation of trabecular meshwork function and IOP. The expression level of TIPARP was elevated in patients with POAG. Downregulation or inhibition of TIPARP could elevate IOP by increasing ECM deposition, producing CLAN and increasing cell adhesion. Thus, TIPARP is a potential target of IOP regulation in POAG.

## Methods

### Study subject enrollment and sample collection

All procedures of this study were approved by the Institutional Review Board of the Eye & ENT Hospital of Fudan University. Informed consent was obtained from all participants. All patients with POAG and controls recruited in our study met the inclusion/exclusion criteria described in our previous study^32^. In brief, the inclusion criteria for patients with POAG were as follows: (1) IOP > 21 mmHg; (2) glaucomatous visual field defects and glaucomatous optic disc damage; (3) open anterior chamber angle; (4) absence of any secondary glaucoma; (5) absence of other ocular diseases that could severely affect visual acuity; (6) brain/orbit magnetic resonance imaging showing no compression lesions. Inclusion criteria for controls were as follows: (1) absence of a history of glaucoma or elevated IOP; (2) absence of very narrow angle; (3) a vertical cup-disc ratio of ≤ 0.5; (4) absence of ocular disorders that could severely affect the visual acuity or visual field; (5) absence of a family history of glaucoma. Trabecular meshwork tissues of the patients with POAG were collected from trabeculectomy surgeries, and control eyeball tissues were obtained from the Eye Bank of the Eye & ENT Hospital, Fudan University. Trabeculectomy for patients with POAG was performed by an experienced ophthalmologist to ensure that trabecular meshwork tissue was included. The trabecular meshwork from the control donor eyes was isolated using a similar method to ensure that the tissues of the two groups had a similar composition. Trabecular meshwork tissues from ten patients with POAG were pooled for protein extraction. Trabecular meshwork tissues from four control donor eyes were pooled for protein extraction. The expression of target proteins in outflow tissue was measured by Western blot. Another group of patients with POAG (*n* = 32) and controls (*n* = 35) were enrolled to test *TIPARP* expression in blood.

### Cell culture and characterization

One strain of primary HTM cells, purchased from ScienCell (Carlsbad, CA), was cultured in Trabecular Meshwork Cell Medium (TMCM, Cat. #6591) at 37 °C under 5% CO_2_, according to the manufacturer’s instructions. For HTM cell characterization, dexamethasone (500 nM) was added to the HTM cell medium for 5 days. The control group was cultured without dexamethasone. The expression of myocilin was measured using both Western blot analyses and immunocytochemistry.

### Western blot analysis

Proteins were extracted from tissues or cells using RIPA lysis buffer (Beyotime). After centrifugation, the supernatant was mixed with 5X loading buffer (Beyotime) and boiled. The denatured proteins were separated by 4–12% or 4–20% SDS polyacrylamide gel (GenScript) and electrophoretically transferred to PVDF membranes (Millipore). The membranes were blocked with 5% nonfat dry milk for 1 h and probed with antibodies against TIPARP (1:100, Abcam ab170817 for Fig. 2; 1:500 ab84664 for the others), GAPDH (1:1000, Cell Signaling Technology #5174), β-actin (1:1000, Cell Signaling Technology #3700), collagen type I (1:1000, Proteintech No. 14695-1-AP), collagen type IV (1:1000, Proteintech No. 55131-1-AP), fibronectin (1:1000, Proteintech No. 15613-1-AP), and α-SMA (1:1000, Abcam ab7817). The membranes were then incubated with peroxidase-linked goat anti-mouse/rabbit IgG secondary antibody (1:3000, Yeasen). Signals were captured on a BioSpectrum imaging system (Ultra-Violet Products) or Kodak Molecular Imaging Software (Kodak).

### Immunofluorescence analyses

Immunofluorescence labeling was performed on human tissues, HTM cells, and siRNA-transfected or RBN-2397-treated HTM cells. Human tissue sections were obtained from donated human eyeballs from the eye bank, fixed with formalin and embedded in paraffin. After deparaffinization and antigen retrieval, the sections were blocked and permeabilized with 5% bovine serum albumin (Beyotime) and 0.1% Triton X-100 (Sigma-Aldrich) for 2 h at room temperature. The sections were incubated with primary anti-TIPARP antibody (1:50, Abcam ab170817) overnight at 4 °C, washed three times in phosphate buffer saline, and then incubated in Alexa Fluor-555 or Alexa Fluor-488 goat anti-rabbit secondary antibody (1:500, Invitrogen/Thermo Fisher) for 2 h at room temperature. Nuclei were visualized by DAPI (4′,6-diamidino-2-phenylindole; Sigma-Aldrich) counterstaining.

HTM cells, which grew on 0.1% gelatin-coated glass coverslips in 24-well plates, were fixed with 4% paraformaldehyde for 15 min and then blocked and permeabilized with 5% bovine serum albumin and 0.1% Triton X-100 for 1 h at room temperature. Then, the coverslips were incubated with anti-TIPARP (1:50, Abcam ab170817) and anti-vinculin (1:150, Abcam ab129002) antibodies overnight at 4 °C and Alexa Fluor-488 or Alexa Fluor-555 goat anti-rabbit secondary antibody (1:500, Invitrogen/Thermo Fisher) for 2 h at room temperature. TRITC-phalloidin Atto 565 (Sigma-Aldrich 94072) was used for F-actin staining for 40 min at room temperature. DAPI staining was performed at room temperature for 5 min.

All primary antibodies, secondary antibodies and DAPI were diluted in phosphate buffer saline containing 1% bovine serum albumin and 0.1% Triton X-100. An inverted confocal microscope (Leica) was used for imaging. The minimum structure of CLAN was defined as three hubs connected by three actin spokes^33,34^. At least three imaging fields were evaluated in each group. The ratio of the number of cells containing CLAN structures to the total number of cells was evaluated^34^.

### RNA isolation and quantitative real-time polymerase chain reaction (qRT‒PCR)

Total RNA was extracted from whole blood using a RNeasy Mini Plus Kit (Qiagen), and total RNA from HTM cells was extracted using an EZ-press RNA purification kit (EZ-Bioscience) according to the manufacturer’s protocol. The RNA concentration was measured using a NanoDrop spectrophotometer. Reverse transcription was performed using a reverse transcription kit (TaKaRa). *TIPARP* mRNA expression was measured by a SYBR Green quantitative real-time PCR kit (TaKaRa) according to the manufacturer’s protocol and analyzed on a ViiA 7 Real-Time PCR System (Life Technologies) or CFX96 Real-Time PCR System (Bio-Rad Laboratories). The primer sequences used were as follows: human *TIPARP* primer F 5’GGCAGATTTGAATGCCATGA3’, primer R 5’TGGACAGCCTTCGTAGTTGGT3’; human *β-actin* primer F 5’TTGTTACAGGAAGTCCCTTGCC3’, primer R 5’ATGCTATCACCTCCCCTGTGTG3’. The relative expression level of mRNA was normalized to that of the reference gene *β-actin*. The results were calculated by the comparative CT method (2^-ΔΔCT^).

### Lentivirus transduction and siRNA transfection in HTM cells

HTM cells were transduced with lentivirus-GFP-TIPARP to upregulate the expression of *TIPARP*. Lentivirus-GFP was used as a control. Lentivirus-GFP-TIPARP and lentivirus-GFP were produced by Hanheng Company (Shanghai, China). According to the manufacturer’s instructions, lentivirus-GFP-TIPARP and lentivirus-GFP were diluted in complete medium containing 8 µg/mL polybrene at a final multiplicity of infection (MOI) of 15. For knockdown of *TIPARP* expression, HTM cells were transfected with siRNA specific to human *TIPARP* (*TIPARP* siRNA) using Lipofectamine 2000 (Invitrogen/Thermo Fisher) according to the manufacturer’s instructions. The control cells were transfected with scramble control siRNA (scrambled siRNA). *TIPARP* siRNA and control siRNA were designed and produced by GenePharma Biological Company (Shanghai, China). Quantitative real-time PCR and western blot were performed to measure TIPARP expression after upregulation or downregulation.

### RNA-seq of HTM cells with *TIPARP* upregulation

To determine the possible effects of *TIPARP*, we performed RNA-seq in *TIPARP*-upregulated HTM cells. After transduction with lentivirus-GFP-TIPARP or lentivirus-GFP for 96 h, the HTM cells were collected for RNA isolation. TRIzol reagent was used for total RNA extraction according to the manufacturer’s protocol. RNA purification, quantification and integrity were evaluated using a NanoDrop 2000 spectrophotometer (Thermo Scientific) and an Agilent 2100 Bioanalyzer (Agilent Technologies). The libraries were constructed by a TruSeq Stranded mRNA LT Sample Prep Kit (Illumina) and sequenced on an Illumina Novaseq 6000 platform. After the raw reads obtained by sequencing were filtered, the clean reads were mapped to the human genome (GRCh38) using HISAT2^35^. The FPKM^36^ of each gene was calculated by Cufflinks^37^, and the read counts of each gene were obtained by HTSeq-count^38^. The DESeq2 was used for differential expression analysis^39^. The threshold for significantly differential expression was q value < 0.05 and fold change > 2 or fold change < 0.5. Hierarchical cluster analysis of differentially expressed genes was used to show the expression pattern of genes in different samples and groups. Gene Ontology enrichment and KEGG pathway enrichment analyses of differentially expressed genes were performed using R^40^.

### Cell contraction assay

HTM cell contraction was tested by a cell contraction assay kit (Cell Biolabs, Inc.) following the manufacturer’s instructions. In brief, HTM cells at a density of 2 ×10^6^ cells/mL were mixed with collagen gel and added to a 24-well plate. After 1 h of incubation at 37 °C for polymerization, the culture medium was added to each well of a plate. After 48 h of incubation, the edge of the gel was carefully detached using a syringe needle. After 13 h, the gel area did not change, and natural contraction was observed. Then, 1 ml of the TIPARP small-molecule inhibitor RBN-2397 (1 μM) was added to the medium. The images were captured at 24 h. The gel area was calculated using ImageJ.

### Animals

All animal experiments were conducted under the ARVO statement for the Use of Animals in Ophthalmic and Vision Research. The experimental protocols were approved by the Institutional Review Board of the Eye & ENT Hospital of Fudan University. *Sprague–Dawley* rats and *C57BL/6* *J* mice were bred and housed in standard cages with a 12 h-12 h light-dark cycle. Male rats aged approximately 8 weeks and weighing 180–200 g were used. Male mice aged 8–10 weeks were used.

### Eye perfusion

For determination of the effect of IOP elevation on TIPARP expression, mouse eyes were perfused with phosphate buffer saline at constant pressures of 6 and 18 mmHg for 4 h^41^. After perfusion, conventional outflow tissues of mice were dissected using an established method^42^. Outflow tissues from three eyes were pooled for protein extraction, and TIPARP expression was measured by Western blot.

### Subconjunctival injection and IOP measurements

All rats were deeply anaesthetized intraperitoneally with ketamine (80 mg/kg) and xylazine (5 mg/kg). Then, 25 μL of 10 µM RBN-2397 was slowly injected under the upper and lower conjunctiva of the eye respectively with a 33-gauge syringe (25 μL volume; Hamilton Company). As a vehicle control, 0.1% dimethylsulfoxide in phosphate buffer saline was injected into the contralateral eye. The injection was repeated on Day 7. IOP measurements were performed before subconjunctival injection and every day after injection. IOP was measured using rebound tonometry (TonoLab, ICare, Espoo, Finland) between 10:00 a.m. and 12:00 p.m. in live animals. Conventional outflow tissues of rats were dissected on Day 14. Outflow tissues from two eyes were pooled for protein extraction. The expression of target proteins in outflow tissue was measured by Western blot.

### Statistics and reproducibility

Statistical analyses were performed with SPSS 20 (IBM-SPSS, Chicago, IL, USA). All experiments were repeated at least three times. Numerical data were presented as the mean ± standard deviation. A *t*-test was used for analysis if the data were normally distributed. The Mann‒Whitney U test was used for analysis if data were not normally distributed. The statistically significant *P* value was set as 0.05.

### Reporting summary

Further information on research design is available in the Nature Portfolio Reporting Summary linked to this article.

## Supplementary information

Supplementary Information

Description of Additional Supplementary Files

Supplementary Data 1

Reporting Summary

## Data Availability

The source data for all the graphs in main figures are provided in Supplementary Data 1. Uncropped western blot images are provided in Supplementary Fig. 4. The RNA sequencing data that support the findings of this study have been deposited in Gene Expression Omnibus (GEO) and are accessible through the GEO Series accession number GSE218153.
