# Supplementary material for: TIPARP is involved in the regulation of intraocular pressure
Source: Commun Biol. 2022 Dec 19;5:1386. doi: 10.1038/s42003-022-04346-0 (PMC9763400; doi:10.1038/s42003-022-04346-0)
Supplement: Supplementary file 1 — Supplementary Information [file 42003_2022_4346_MOESM1_ESM.pdf]

Supplementary Information for

TIPARP is involved in the regulation of intraocular pressure

Youjia Zhang[1,2#]; Maomao Song[1,2#]; Yingwen Bi[3]; Yuan Lei[1,2*]; Xinghuai Sun[1,2,4*]; Yuhong Chen[1,2*]

1. Department of Ophthalmology & Visual Science, Eye & ENT Hospital, Shanghai Medical College, Fudan University, Shanghai 200031, China
2. NHC Key Laboratory of Myopia, Chinese Academy of Medical Sciences, and Shanghai Key Laboratory of Visual Impairment and Restoration, Fudan University, Shanghai 200031, China
3. Department of Pathology, Eye & ENT Hospital, Shanghai Medical College, Fudan University, Shanghai 200031, China
4. State Key Laboratory of Medical Neurobiology and MOE Frontiers Center for Brain Science, Institutes of Brain Science, Fudan University, Shanghai 200032, China

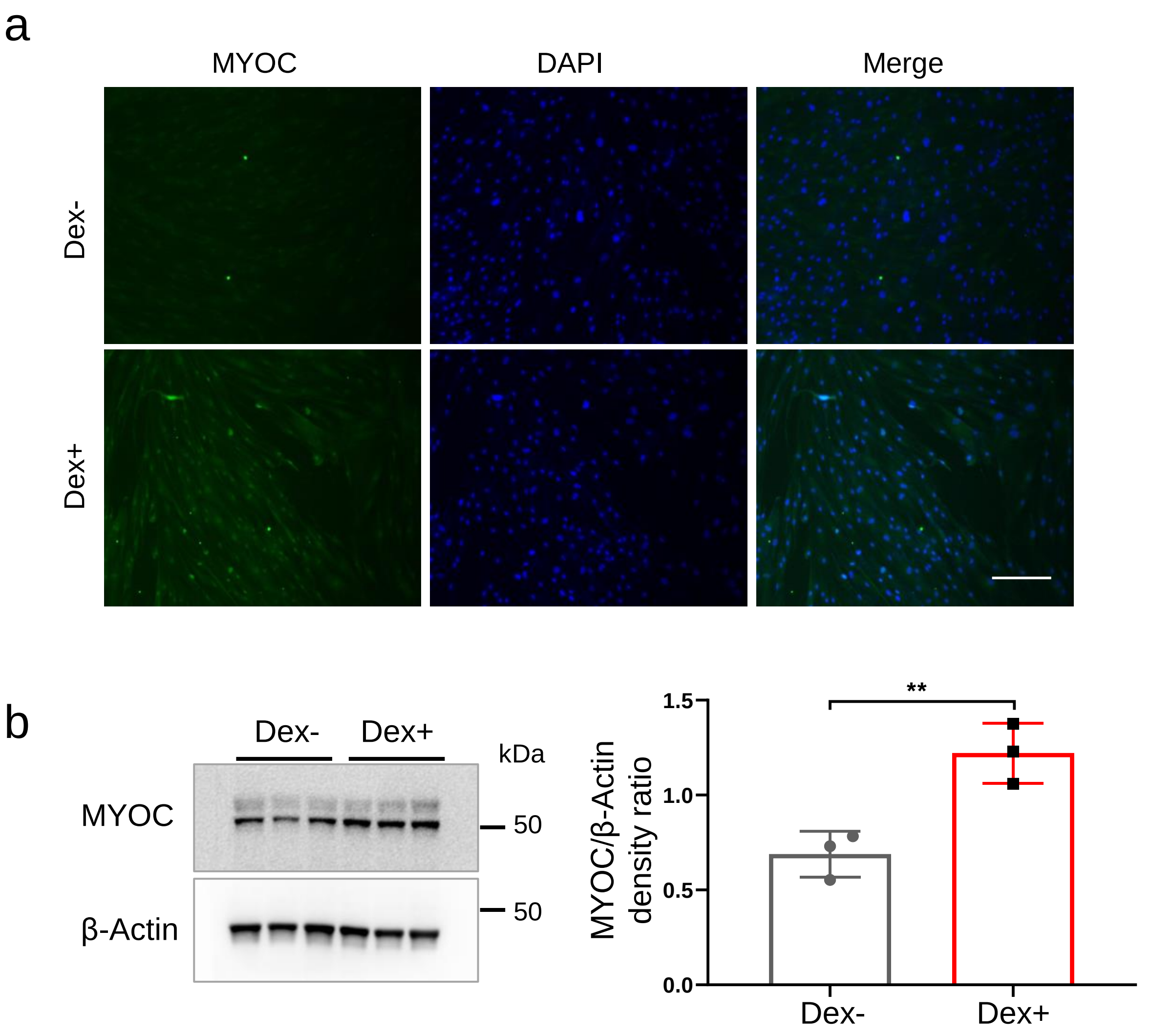

Supplementary Figure 1: The human trabecular meshwork (HTM) cells identification. (a) Immunofluorescence staining showed increase of myocilin in dexamethasone (Dex) treated HTM cells. (b) Western blot and quantitative analyses showed the myocilin expression increased in Dex treated HTM cells than that in HTM cells without Dex treated (n=3, t-test). Data are presented as the means ± standard deviations. **$p<0.01$, scale bar = 50 μm. Abbreviations: Dex, dexamethasone; MYOC, myocilin.

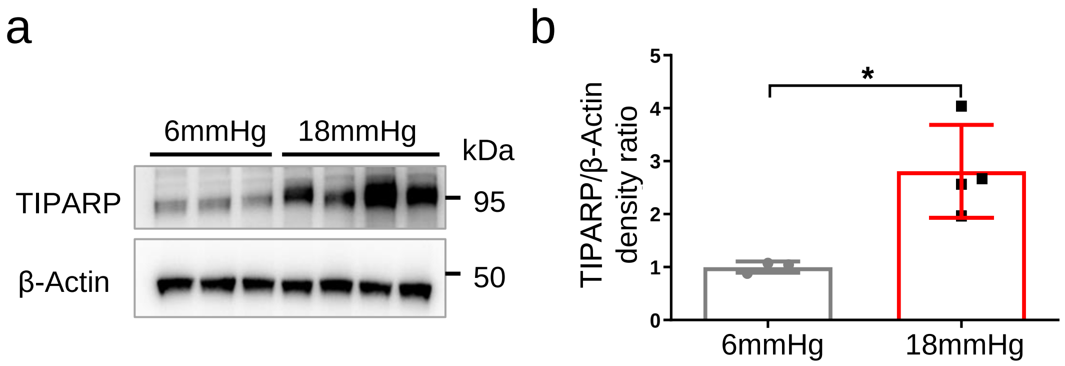

Supplementary Figure 2: TIPARP expression in outflow tissues of mouse eyes upregulated under high pressure perfusion. To characterize TIPARP expression under elevated IOP, the enucleated mouse eyes were perfused at constant pressures. The expression of TIPARP was upregulated in 18 mmHg perfusion compared with that in 6 mmHg perfusion (n = 3 in 6 mmHg group and n = 4 in 18 mmHg group, t-test). Data are presented as the means ± standard deviations.*$p<0.05$.

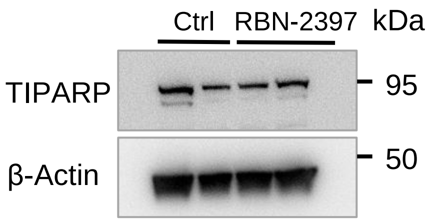

Supplementary Figure 3: TIPARP inhibitor RBN-2397 did not change the TIPARP expression. Western blot showed the expression of TIPARP did not change in HTM cells with and without RBN-2397 treatment.

Figure 1a

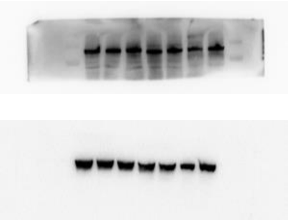

Figure 2b

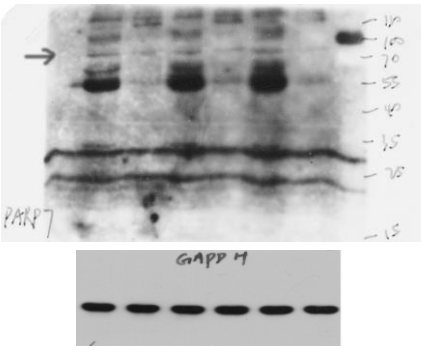

Figure 3c

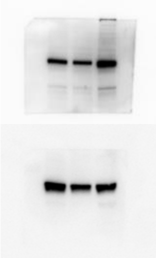

Figure 4c

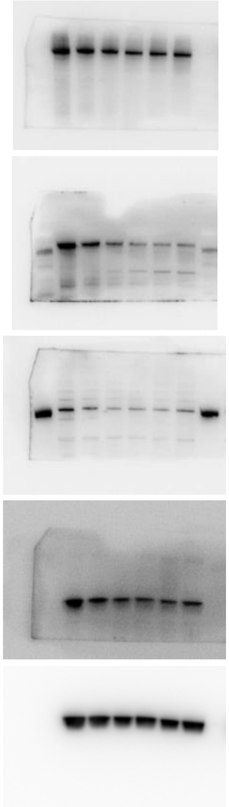

Figure 5c

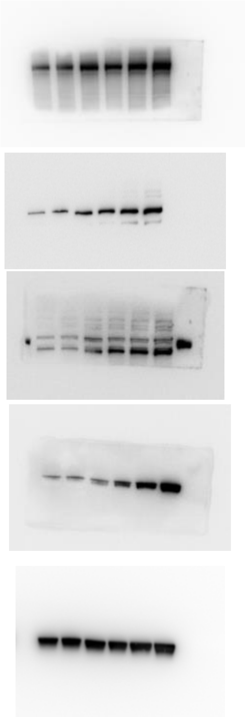

Figure 6b

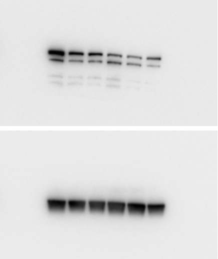

Figure 6c

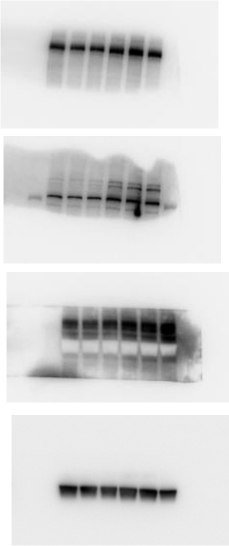

Figure 6c

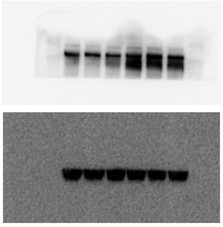

Figure 7c

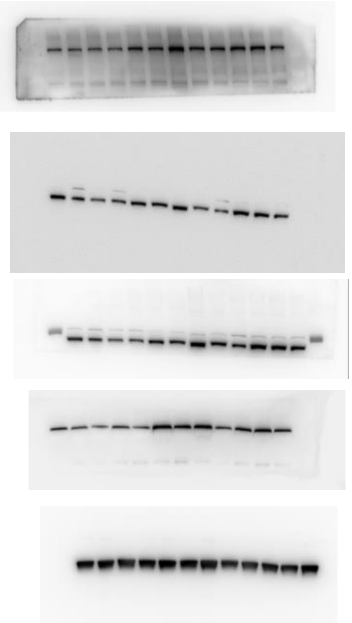

Supplementary Figure 1b

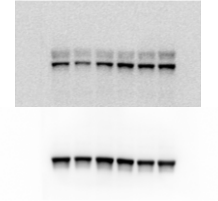

Supplementary Figure 2a

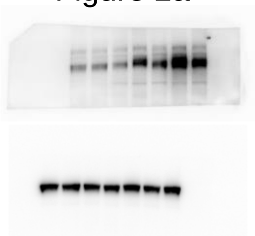

Supplementary Figure 3

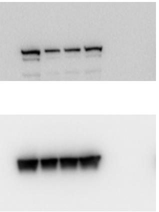

Supplementary Figure 4. uncropped western blots.
