# Supplementary material for: TIPARP is involved in the regulation of intraocular pressure
Source: Commun Biol. 2022 Dec 19;5:1386. doi: 10.1038/s42003-022-04346-0 (PMC9763400; doi:10.1038/s42003-022-04346-0)
Supplement: Supplementary file 2 — Description of Additional Supplementary Files [file 42003_2022_4346_MOESM2_ESM.pdf]

Description of Additional Supplementary Files

File name: Supplementary Data 1

Description: The source data behind the graphs in the paper
