# Supplementary material for: TIPARP is involved in the regulation of intraocular pressure
Source: Commun Biol. 2022 Dec 19;5:1386. doi: 10.1038/s42003-022-04346-0 (PMC9763400; doi:10.1038/s42003-022-04346-0)
Supplement: Supplementary file 4 — Reporting Summary [file 42003_2022_4346_MOESM4_ESM.pdf]

nature portfolio

Corresponding author(s): Yuhong Chen, Xinghuai Sun, Yuan Lei

Last updated by author(s): Nov 18, 2022

Reporting Summary

Nature Portfolio wishes to improve the reproducibility of the work that we publish. This form provides structure for consistency and transparency in reporting. For further information on Nature Portfolio policies, see our Editorial Policies and the Editorial Policy Checklist.

Statistics

For all statistical analyses, confirm that the following items are present in the figure legend, table legend, main text, or Methods section.

n/a	Confirmed	
☐	☒	The exact sample size (*n*) for each experimental group/condition, given as a discrete number and unit of measurement
☐	☒	A statement on whether measurements were taken from distinct samples or whether the same sample was measured repeatedly
☐	☒	The statistical test(s) used AND whether they are one- or two-sided<br>*Only common tests should be described solely by name; describe more complex techniques in the Methods section.*
☒	☐	A description of all covariates tested
☐	☒	A description of any assumptions or corrections, such as tests of normality and adjustment for multiple comparisons
☐	☒	A full description of the statistical parameters including central tendency (e.g. means) or other basic estimates (e.g. regression coefficient) AND variation (e.g. standard deviation) or associated estimates of uncertainty (e.g. confidence intervals)
☐	☒	For null hypothesis testing, the test statistic (e.g. *F*, *t*, *r*) with confidence intervals, effect sizes, degrees of freedom and *P* value noted<br>*Give P values as exact values whenever suitable.*
☒	☐	For Bayesian analysis, information on the choice of priors and Markov chain Monte Carlo settings
☒	☐	For hierarchical and complex designs, identification of the appropriate level for tests and full reporting of outcomes
☒	☐	Estimates of effect sizes (e.g. Cohen's *d*, Pearson's *r*), indicating how they were calculated

Our web collection on statistics for biologists contains articles on many of the points above.

Software and code

Policy information about availability of computer code

Data collection	No software was used to collect the data.
Data analysis	The differential expression detected by RNA-Seq was analyzed by DESeq2. Gene Ontology enrichment and KEGG pathway enrichment analyses of differentially expressed genes were performed using R. Statistical analyses were performed with SPSS 20 (IBM-SPSS, Chicago, IL, USA).

For manuscripts utilizing custom algorithms or software that are central to the research but not yet described in published literature, software must be made available to editors and reviewers. We strongly encourage code deposition in a community repository (e.g. GitHub). See the Nature Portfolio guidelines for submitting code & software for further information.

Data

Policy information about availability of data

All manuscripts must include a data availability statement. This statement should provide the following information, where applicable:

- Accession codes, unique identifiers, or web links for publicly available datasets
- A description of any restrictions on data availability
- For clinical datasets or third party data, please ensure that the statement adheres to our policy

All data generated or analysed during this study are included in this published article (and its supplementary information files).

Human research participants

Policy information about studies involving human research participants and Sex and Gender in Research.

Reporting on sex and gender	This study was not a research on human population.
Population characteristics	This study was not a research on human population.
Recruitment	All patients with POAG and controls recruited in Eye & ENT Hospital of Fudan University. All patients with POAG and controls recruited in our study met the inclusion/exclusion criteria described in our previous study (Chen et al. Nat Genet 2014). The inclusion criteria for patients with POAG were as follows: 1) IOP $>$ 21 mmHg; 2) glaucomatous visual field defects and glaucomatous optic disc damage; 3) open anterior chamber angle; 4) absence of any secondary glaucoma; 5) absence of other ocular diseases that could severely affect visual acuity; 6) brain/orbit magnetic resonance imaging showing no compression lesions. Inclusion criteria for controls were as follows:1) absence of a history of glaucoma or elevated IOP; 2) absence of very narrow angle; 3) a vertical cup-disc ratio of $\leq$ 0.5; 4) absence of ocular disorders that could severely affect the visual acuity or visual field; 5) absence of a family history of glaucoma.
Ethics oversight	the Institutional Review Board of the Eye & ENT Hospital of Fudan University

Note that full information on the approval of the study protocol must also be provided in the manuscript.

Field-specific reporting

Please select the one below that is the best fit for your research. If you are not sure, read the appropriate sections before making your selection.

[x] Life sciences   [ ] Behavioural & social sciences   [ ] Ecological, evolutionary & environmental sciences

For a reference copy of the document with all sections, see nature.com/documents/nr-reporting-summary-flat.pdf

Life sciences study design

All studies must disclose on these points even when the disclosure is negative.

Sample size	The formula $(Z\alpha+Z\beta)(Z\alpha+Z\beta)*2*SD*SD/\delta*\delta$ was used to estimate the sample size for the TIPARP expression in the blood samples. SD and δ were set according to our preliminary data. $Z\alpha$ and $Z\beta$ were obtained at $\alpha = 0.05$ and β=0.9. The sample size was calculated as 30 in each group. The actual sample size used in our study (n=35 in control group and n=32 in POAG group) was sufficient to reach the power.
Data exclusions	No data were excluded from analyses.
Replication	Each of our experiments was repeated at least three times independently.
Randomization	This study was not a randomized controlled trial, so randomization was not relevant to our study.
Blinding	This study was not a clinical trial. The main experiments were performed by the first authors without blinding.

Reporting for specific materials, systems and methods

We require information from authors about some types of materials, experimental systems and methods used in many studies. Here, indicate whether each material, system or method listed is relevant to your study. If you are not sure if a list item applies to your research, read the appropriate section before selecting a response.

Materials & experimental systems

n/a	Involved in the study	
[ ]	[x]	Antibodies
[ ]	[x]	Eukaryotic cell lines
[x]	[ ]	Palaeontology and archaeology
[ ]	[x]	Animals and other organisms
[x]	[ ]	Clinical data
[x]	[ ]	Dual use research of concern

Methods

n/a	Involved in the study	
[x]	[ ]	ChIP-seq
[x]	[ ]	Flow cytometry
[x]	[ ]	MRI-based neuroimaging

Antibodies

Antibodies used	TIPARP (Abcam ab170817 lot:GR134520-14); TIPARP (Abcam ab84664 lot: GR3371602-2); vinculin (Abcam ab129002,lot: GR3395452-2) ; β-actin (Cell Signaling Technology #3700 lot: 17); GAPDH (Cell Signaling Technology #5174 lot: 8) collagen type I (Proteintech No. 14695-1-AP,lot: 00090638), collagen type IV (Proteintech No. 55131-1-AP,lot: 00086660), fibronectin (Proteintech No. 15613-1-AP,lot: 00108123), α-SMA (Abcam ab7817,lot: GR3257713-12)
Validation	TIPARP (ab170817, human/mouse, WB/IF); TIPARP (ab84664, human/mouse, WB); vinculin (human, IF) ; β-actin (human/mouse, WB), GAPDH (human/mouse, WB); collagen type I (human/mouse, WB); collagen type IV (human/mouse, WB); fibronectin (human/mouse, WB); α-SMA (human/mouse, WB)

Eukaryotic cell lines

Policy information about cell lines and Sex and Gender in Research

Cell line source(s)	One strain of primary human trabecular meshwork (HTM) cells was purchased from ScienCell (Carlsbad, CA).
Authentication	HTM cell characterization was performed by a classical method. After 5 days of treatment with dexamethasone (DEX), the myocilin expression of HTM was increased according to immunofluorescence (Supplementary Figure 1a) and Western blot analyses (Supplementary Figure 1b).
Mycoplasma contamination	All cell lines tested negative for mycoplasma contamination.
Commonly misidentified lines (See ICLAC register)	No commonly misidentified line was used.

Animals and other research organisms

Policy information about studies involving animals; ARRIVE guidelines recommended for reporting animal research, and Sex and Gender in Research

Laboratory animals	C57BL/6J mice, 6-8 weeks and Sprague–Dawley (SD) rats, 180-200g
Wild animals	The study did not involve wild animals.
Reporting on sex	Male rats and mice were used.
Field-collected samples	The study did not involve samples collected from field.
Ethics oversight	the Institutional Review Board of the Eye & ENT Hospital of Fudan University

Note that full information on the approval of the study protocol must also be provided in the manuscript.
